# A Commendable Reform

**Published:** 1909-09

**Authors:** 


### A COMMENDABLE REFORM.

That there are too many medical colleges and that too many doctors are being made, is manifest, and merging of colleges and elevating the standard is the order of the day. We note such consolidation in Cincinnati, Louisville and Nashville. My, what a host of "professors" are out of employment! The most remarkable and latest consolidation is that of the two old schools of Nashville, the following full account of which I reproduce from the *Southern Practitioner*, edited by the Dean of the Medical

Profession South, the grand old Confederate surgeon and long-time teacher of medicine, Dr. Deering J. Roberts. He says:

The union of two of the oldest universities in the Southwest in support of a joint medical college is the most important recent event in Southern medical education. After more than a century of brilliant achievements in the cause of higher education in the South, these two State institutions have combined their medical faculties, laboratory and hospital equipment and strong financial backing in the formation of one powerful medical college with standard entrance requirements and curriculum. This institution is one of the few in the South not entirely dependent upon receipts from tuition. Many thousands of dollars for increased laboratory equipment have been placed at the immediate disposal of the several departments. The purpose to provide the best fundamental instruction has been fulfilled by the employment of thoroughly scientific laboratory men who will be amply compensated for their services.

The adoption of higher preliminary requirements has become feasible as the result of the great increase in the number of standard high schools throughout the South during the past two years. The University of Tennessee, one of the parties to this union of strength, has by recent act of the Legislature become the head of the public school system of the State. Within a comparatively short time every county in Tennessee will have a fourteen unit high school, whose graduates may receive free tuition in the University with traveling expenses paid by the State, so as to make it equally accessible to students from every county. Students intending to study medicine may so elect their course as to attend the Literary Department at Knoxville three years and apply for their A. B. or B. S. degree at the end of their first year in medicine at Nashville, and after three more years in the Medical Department receive their M. D. degree. The agreement of combination between the two universities anticipates that a similar arrangement will be made by the University of Nashville. Thus the combined degree, A. B., M. D. or B. S., M. D., becomes possible after seven years of study, three in the literary and four in the medical.

Practically all of the instruction with the exception of the clinical will be given in the building formerly occupied by the Medical Department of the University of Nashville. The greater part of the senior work will be given in the college hospital on Broadway in the building formerly used by the University of Tennessee as its Medical Department. This large four-story building already possessing an amphitheater of remarkable suitability for observance of clinical operations by students is being remodeled and thoroughly equipped to accommodate sixty or seventy beds, and will be in operation by the first day of September. Although ample provision will be made for charity patients, particularly desirable accommodations are provided for those able to pay. The equipment will be first class and worthy to be installed in the large six-story hospital to be erected on the old medical college site, which is part of the original grant from North Carolina in 1785. This spot is dear to the hearts of thousands of alumni who studied under Lindsley, the elder Eve, Bowling, T. L. Maddin, W. T. Briggs and others of equal fame when there were few schools west of the Alleghenies.

